# Silencing DNA methyltransferase 1 (DNMT1) inhibits proliferation, metastasis and invasion in ESCC by suppressing methylation of RASSF1A and DAPK

**DOI:** 10.18632/oncotarget.9866

**Published:** 2016-06-07

**Authors:** Jian Bai, Xue Zhang, Kai Hu, Bangqing Liu, Haiyong Wang, Angui Li, Feng Lin, Lifei Zhang, Xiaolin Sun, Zhenzong Du, Jianfei Song

**Affiliations:** ^1^ Department of Thoracic & Cardiovascular Surgery, The Second Affiliated Hospital of Guilin Medical University, Guilin, China; ^2^ Department of ICU, The Second Affiliated Hospital of Guilin Medical University, Guilin, China; ^3^ Department of Thoracic & Cardiovascular Surgery, The Affiliated Hospital of Guilin Medical University, Guilin, China; ^4^ Current address: Department of Thoracic & Cardiovascular Surgery, The Second Affiliated Hospital of Guilin Medical University, Lingui District, Guilin, China

**Keywords:** ESCC, DNA methyltransferase 1, methylation, RASSF1A, DAPK

## Abstract

Our previous study showed DNMT1 is up-regulated in esophageal squamous cell carcinoma (ESCC), which is associated with methylation of tumor suppressors. In the current study, we investigate the role of DNMT1 in ESCC. We found silencing DNMT1 inhibited proliferation, metastasis and invasion of three different ESCC cells, K150, K410 and K450. We also found silencing DNMT1 induced G1 arrest and cell apoptosis in K150, K410 and K450 cells. In vivo study showed silencing DNMT1 suppressed tumor growth in nude mice. In addition, silencing DNMT1 increased expression of tumor suppressor genes, RASSF1A and DAPK, in ESCC cells and ESCC xenograft in nude mice. Moreover, silencing DNMT1 decreased methylation in promoter of RASSF1A and DAPK. In conclusion, our data demonstrated that silencing DNMT1 inhibits proliferation, metastasis and invasion in ESCC by suppressing methylation of RASSF1A and DAPK.

## INTRODUCTION

Esophageal squamous cell carcinoma (ESCC) is one of the most common malignancies in worldwide. The incidence of ESCC in China is much higher than other countries. Figuring out the molecular mechanisms of carcinogenesis, metastasis and invasion in ESCC will help improving efficiency of ESCC therapy. Although numerous efforts had been made, the mechanism of ESCC is not fully understood yet.

Recently, epigenetic silencing of tumor suppressor genes has been demonstrated as one of the most important mechanisms contributes to inactivation of tumor suppressor genes in cancer. Promoter methylation of tumor suppressor genes is a critical early step in carcinogenesis including that of ESCC. Our previous study indicates that the status of promoter methylation changes following the progression of ESCC. P16 methylation is a frequent and early event in ESCC. Methylation of MLH1 was associated with advanced stage ESCC. The aberrant methylation of tumor suppressor genes has also been used as a predictor of the clinical outcome following a curative resection of ESCC. Promoter methylation of APC and FHIT has been associated with reduced survival in ESCC patients after esophagectomy.

RASSF1A is member of Ras association (RalGDS/AF-6) domain family [1]. Loss or altered expression of RASSF1A has been associated with the pathogenesis of a variety of cancers, which suggests the tumor suppressor function of this gene. The inactivation of RASSF1A was found to be correlated with the hypermethylation of its CpG-island promoter region [2–5]. Our previous study indicates methylation of RASSF1A associates with expression of DNMT1 [6] in ESCC. Death-associated protein kinase (DAPK) is an intracellular protein that mediates cell death by its serine/threonine kinase activity, and transmits apoptotic cell death signals [7, 8]. DAPK promoter methylation is associated with carcinogenesis [9].

DNA methyltransferase 1 (DNMT1) plays an important role in the establishment and regulation of tissue-specific patterns of methylated cytosine residues [10, 11]. DNMT1 mediated epigenetic silencing of tumor suppressor genes contributes to the progression of cancer [12, 13], which makes DNMT1 the potential target of cancer therapy. Several studies report that RNAi mediated depletion of DNMT1 or microRNA mediated suppression of DNMT1 restores tumor suppressor genes expression through the reversal of DNA hypermethylation [14–17]. Our previous study show DNMT1 is highly expressed in ESCC specimens [6]. However, the function of DNMT1 in ESCC has not been reported.

In the present study, we investigate the effects of silencing DNMT1 on proliferation, metastasis and invasion of ESCC. The molecular mechanism underlying effects of silencing DNMT1 on ESCC is also examined by focusing on the methylation status of two tumor suppressor genes, RASSF1A and DAPK.

## RESULTS

### Silencing DNMT1 inhibits proliferation, metastasis and invasion in ESCC cells

**Figure 1:**
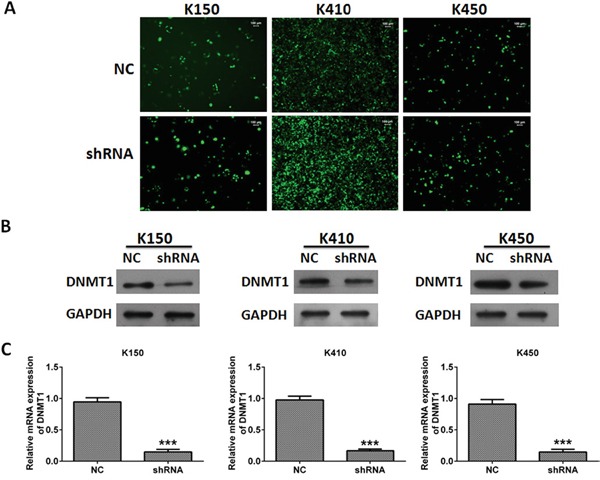
Silencing DNMT1 in ESCC cells **A.** Representative pictures of ESCC cells infected with lentivirus. Magnification, 20 ×. **B.** The protein expression of DNMT1 in ESCC cells. GAPDH was served as loading control. **C.** The mRNA expression of DNMT1 in ESCC cells. mRNA expression of DNMT1 was normalized to GAPDH. Data represented as means ± SD from three independent experiments. ***, *p* < 0.001.

Our previous study showed DNMT1 overexpressed in ESCC specimens. In order to investigate the role of DNMT1 in ESCC, we examined the effects of silencing DNMT1 on proliferation, metastasis and invasion in ESCC cells. We created three ESCC stable cell lines (K150-shRNA, K410-shRNA and K450-shRNA) which the expression of DNMT1 was suppressed by shRNA targeted DNMT1. Their corresponding control were ESCC cells infected with lentivirus-NC, which containing scramble sequence. To confirm the creation of ESCC stable cells, the mRNA and protein expression of DNMT1 were examined by quantitative real time RT-PCR and western blot, respectively. Our data showed both mRNA and protein expression of DNMT1 in three ESCC stable cell lines was significantly suppressed compare to their controls (Figure 1).

**Figure 2:**
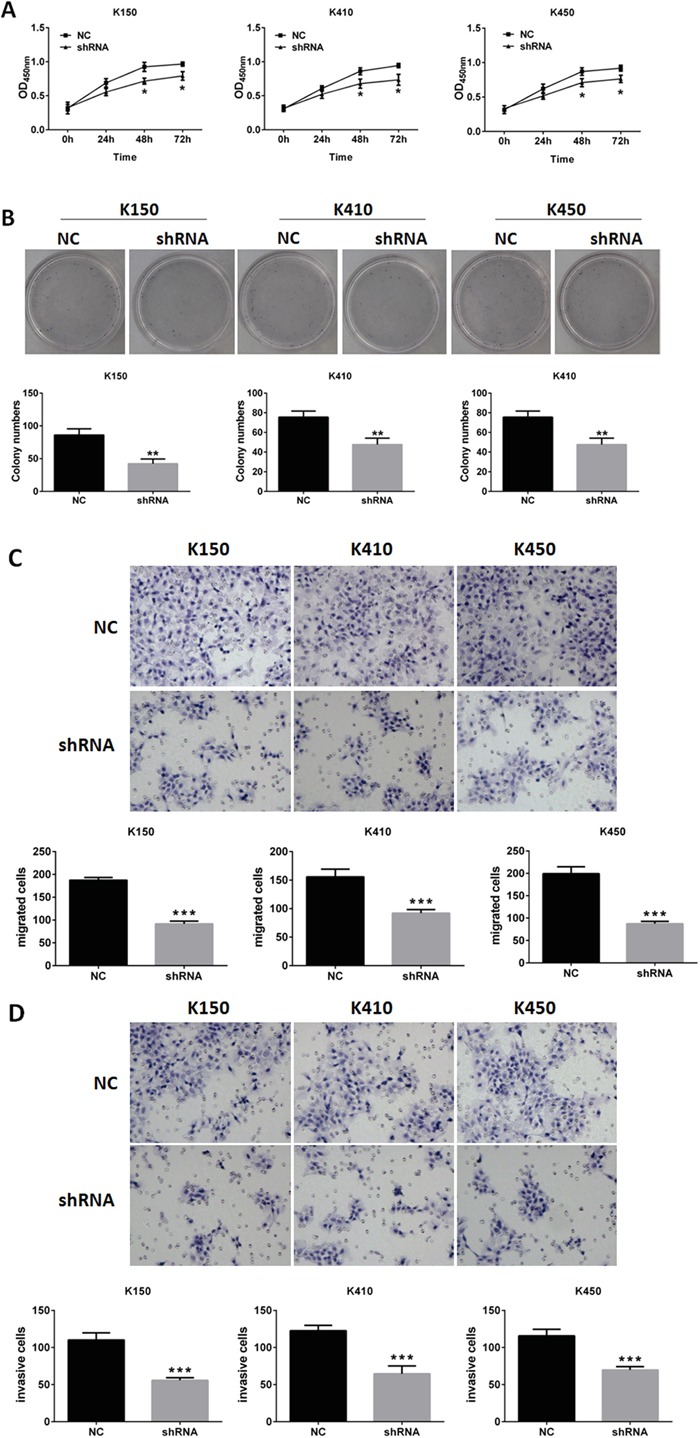
Silencing DNMT1 inhibited proliferation, metastasis and invasion in ESCC cells **A.** MTT assays of ESCC cells. Data represented as means ± SD from three independent experiments. *, *p*< 0.05. **B.** colony formation assays of ESCC cells. Data represented as means ± SD from three independent experiments. **, *p* < 0.01. **C.** metastasis assays of ESCC cells. original magnification, 20 X. Data represented as means ± SD from three independent experiments. ***, *p* < 0.001. **D.** invasion assays of ESCC cells. original magnification, 20 ×. Data represented as means ± SD from three independent experiments. ***, *p* < 0.001.

Cell proliferation assays and colony formation assays were performed to evaluate cell growth and colony formation ability of K150-shRNA, K410-shRNA and K450-shRNA stable cells. We found ESCC stable cells grew much slower and formed less colonies than their corresponding controls (Figure 2A and 2B). In vitro transwell assay was performed to evaluate metastasis and invasion abilities of ESCC stable cells. our data showed metastasis and invasion abilities of ESCC stable cells were much weaker than their corresponding controls (Figure 2C and 2D). These results indicates silencing DNMT1 inhibits proliferation, metastasis and invasion in ESCC cells.

### Silencing DNMT1 induces G1 arrest and apoptosis in ESCC cells

**Figure 3:**
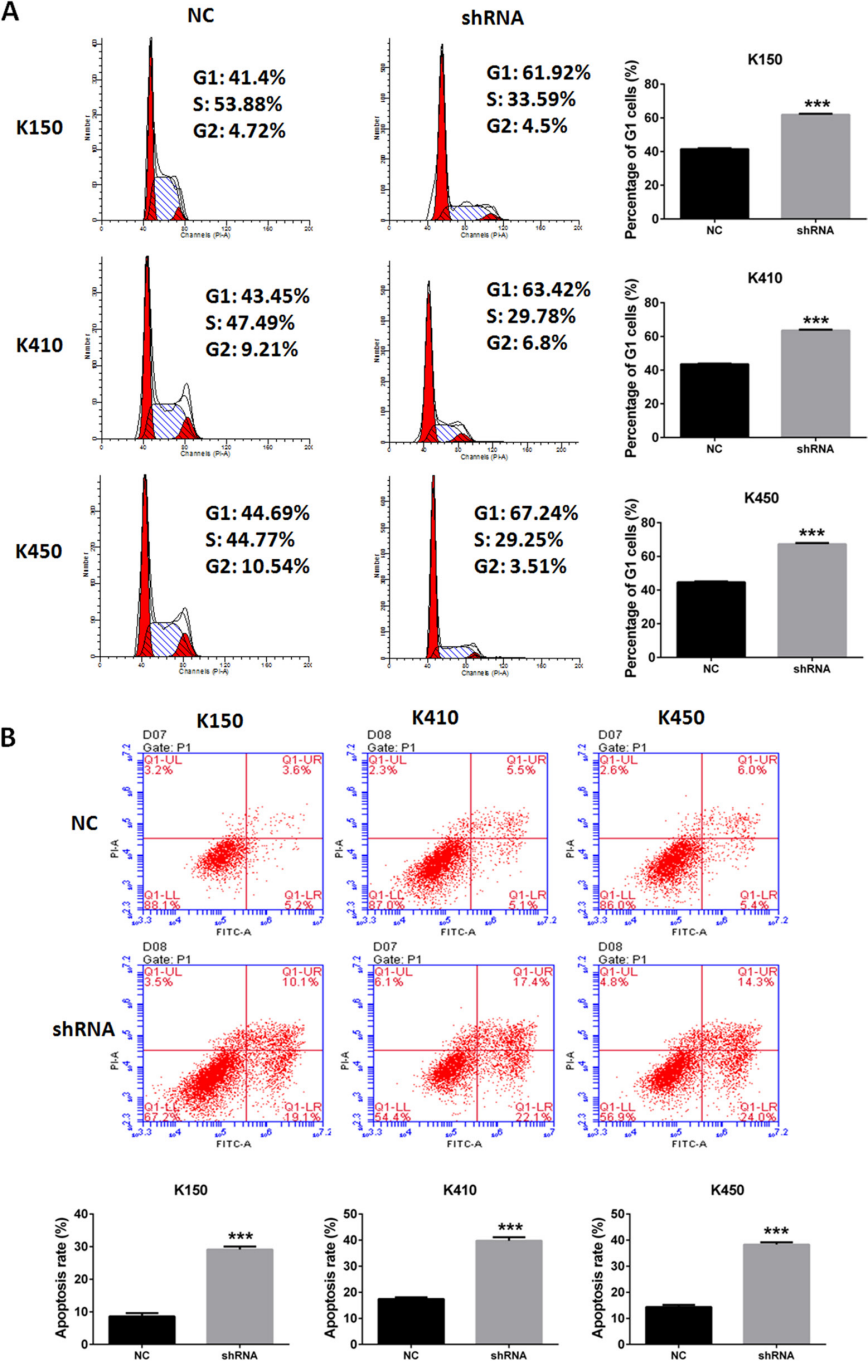
Silencing DNMT1 induced G1 arrest and apoptosis in ESCC cells **A.** Cell cycle analysis of ESCC stable cells. Data represented as means ± SD from three independent experiments. ***, *p* < 0.001. **B.** Apoptosis assays of ESCC stable cells. Data represented as means ± SD from three independent experiments. ***, *p*< 0.001.

In order to further explore the mechanism of inhibitory effects of silencing DNMT1 on ESCC, we performed cell cycle analysis and apoptosis analysis in ESCC stable cells. our data showed the percentage of G1 cells and apoptosis rate was increased in ESCC stable cells compared with their corresponding controls (Figure 3A and 3B). These data suggest that silencing DNMT1 induces G1 arrest and apoptosis in ESCC cells.

### Silencing DNMT1 suppresses tumor growth *in vivo*

**Figure 4:**
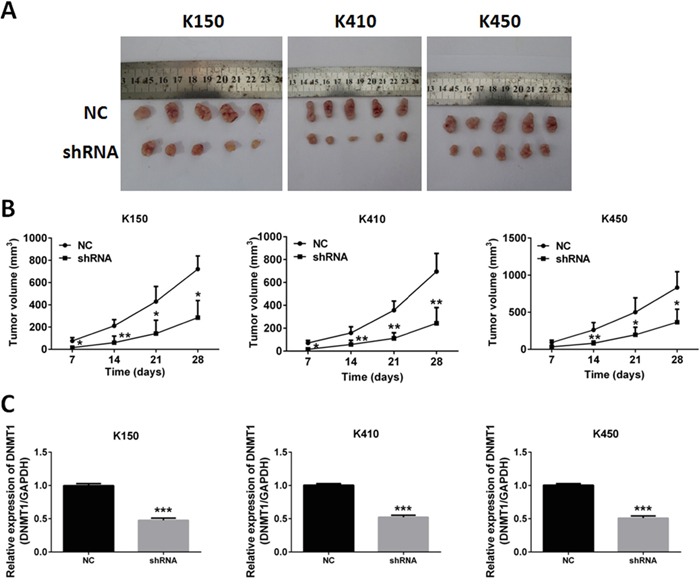
Silencing DNMT1 inhibited tumor growth in nude mice **A.** Tumors isolated from nude mice injected with ESCC stable cells. **B.** Growth curve of Tumors. Data represented as means ± SD. *, *p* < 0.05. **, *p* < 0.01. **C.** expression of DNMT1 in tumors isolated from nude mice. Data represented as means ± SD. ***, *p* < 0.001.

We further verified these findings by examining the effects of silencing DNMT1 on tumor growth in nude mice. K150-shRNA, K410-shRNA and K450-shRNA stable cells were injected subcutaneously into nude mice, respectively. Control groups were injected with K150-NC, K410-NC and K450-NC stable cells. The tumor sizes were recorded at day 7, 14, 21 and 28. We found group K140, group K410 and group K450 generated much smaller tumors than their corresponding controls (Figure 4A and 4B). Quantitative real time RT-PCR was performed to confirm the suppression of DNMT1 in tumors of nude mice (Figure 4C). These data suggest that silencing DNMT1 suppresses tumor growth in vivo.

### Silencing DNMT1 up-regulates expression of RASSF1A and DAPK

**Figure 5:**
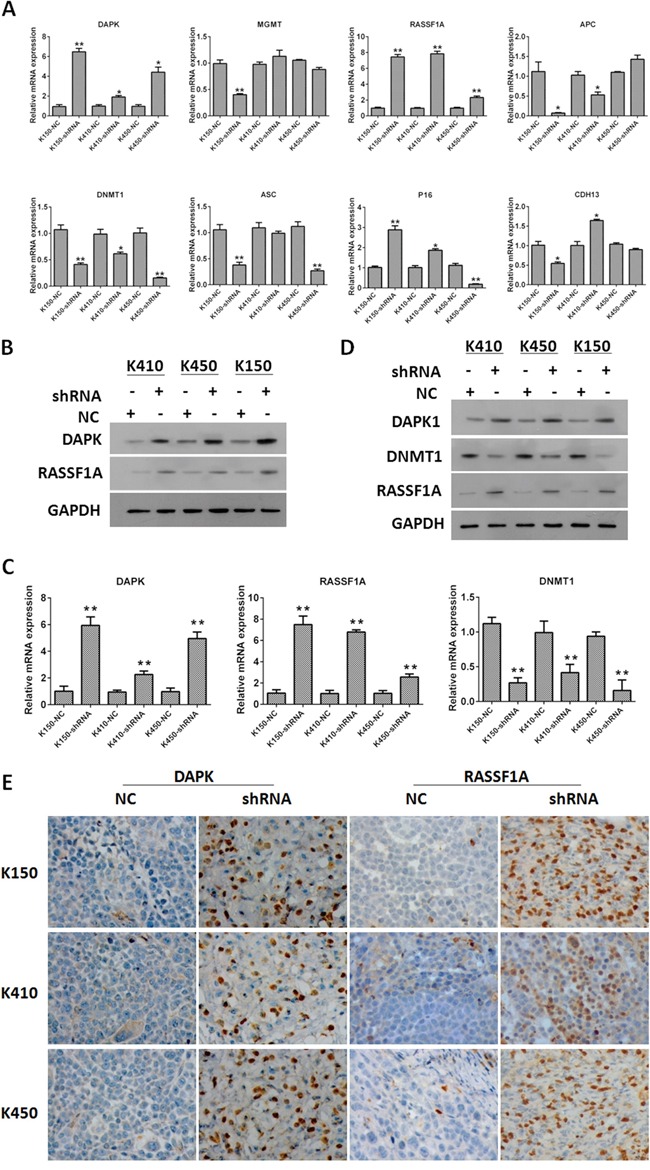
Silencing DNMT1 up-regulated expression of RASSF1A and DAPK **A.** The mRNA expression of DAPK, MGMT, RASSF1A, APC, DNMT1, ASC, P16 and CDH13 in ESCC stable cells. mRNA expression of tumor suppressors was normalized to GAPDH. Data represented as means ± SD. *, *p*< 0.05. **, *p* < 0.01. **B.** The protein expression of DAPK and RASSF1A in ESCC cells. GAPDH was served as loading control. **C.** The mRNA expression of DAPK, RASSF1A and DNMT1 in tumors isolated from nude mice injected with ESCC stable cells. mRNA expression of tumor suppressors was normalized to GAPDH. Data represented as means ± SD. *, *p* < 0.05. **, *p* < 0.01. **D.** The protein expression of DAPK and RASSF1A in tumors isolated from nude mice injected with ESCC stable cells. GAPDH was served as loading control. Data represented as means ± SD. *, *p*< 0.05. **, *p*< 0.01. **E.** IHC staining of DAPK and RASSF1A in tumors isolated from nude mice injected with ESCC stable cells.

In order to demonstrate the molecular mechanisms underlying inhibitory effects of silencing DNMT1 on ESCC cells, we investigated the effect of silencing DNMT1 on expression of tumor suppressor genes. Our data showed mRNA and protein expressions of RASSF1A and DAPK in K150-shRNA, K410-shRNA and K450-shRNA stable cells were significantly higher than those in corresponding controls (Figure 5A and 5B). Similar results were obtained from tumors isolated from nude mice injected with ESCC stable cells (Figure 5C, 5D and 5E). These data suggest that silencing DNMT1 up-regulates expression of RASSF1A and DAPK. This results were verified by rescue experiments by overexpression of RASSF1 and DAPK in DNMT1 knockdown cells (Supplementary Figure S3-S5).

### Silencing DNMT1 suppresses methylation of RASSF1A and DAPK

**Figure 6:**
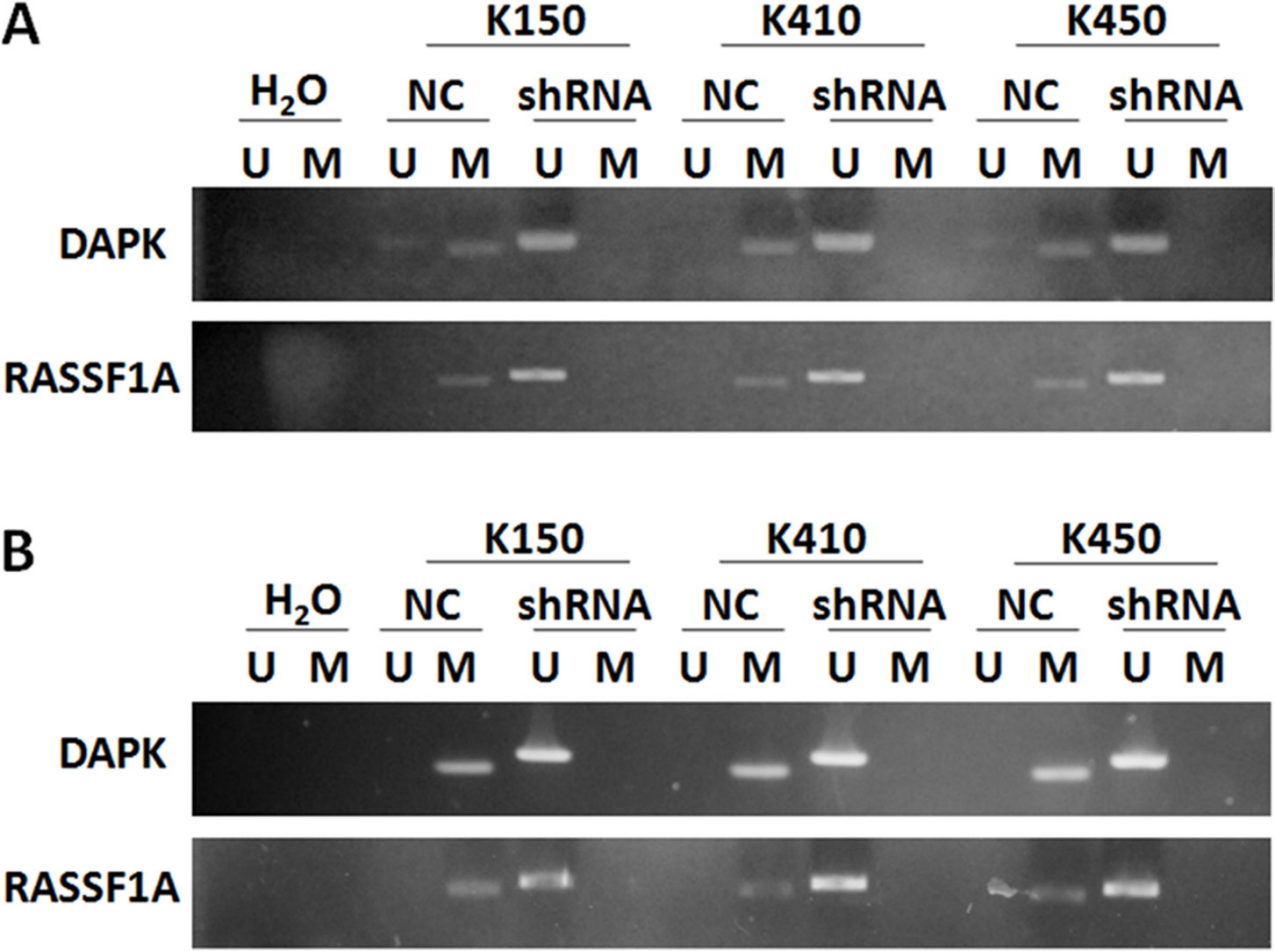
Silencing DNMT1 suppressed methylation in promoter of RASSF1A and DAPK **A.** Methylation status of RASSF1A and DAPK in ESCC stable cells. **B.** Methylation status of RASSF1A and DAPK in tumors isolated from nude mice injected with ESCC stable cells.

**Figure 7:**
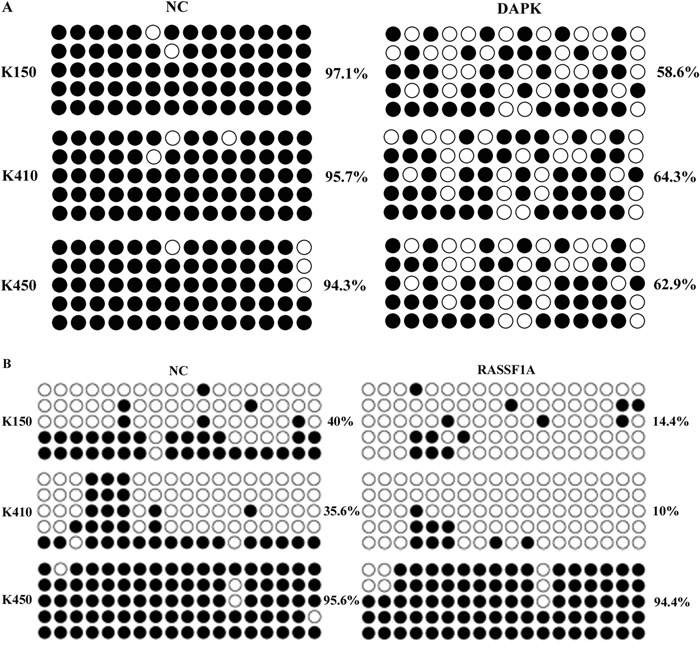
Bisulfite sequencing of the DAPK and RASSF1A CpG island in three ESCC stable cells Open and filled circles represent unmethylated and methylated CpG sites, respectively. Each horizontal row represents a single clone. There are 14 and 18 CpG sites in the region of DAPK and RASSF1A, respectively.

Methylation of tumor suppressor genes, which results in down-regulation of tumor suppressor genes, is one of the mechanisms contribute to ESCC. To further demonstrate molecular mechanisms underlying effects of silencing DNMT1 on RASSF1A and DAPK, we analyzed methylation of RASSF1A and DAPK in ESCC stable cells by MSP and BSP. Our data showed methylation of RASSF1A and DAPK were inhibited in K150-shRNA, K410-shRNA and K450-shRNA stable cells, but not in their corresponding controls (Figure 6A & Figure 7).

This finding was supported by results obtained from tumors isolated from nude mice injected with ESCC stable cells. Methylation of RASSF1A and DAPK were both inhibited in tumors isolated from nude mice injected with ESCC stable cells compared with their corresponding controls (Figure 6B).

## DISCUSSION

DNMT1 is required to maintained CpG methylation and aberrant gene silencing in multiple physical process and diseases, including cell differentiation [18, 19], T cell development and function [20], differentiation and function of stem cells [21–23], systemic lupus erythematosus [24], tumor initiation and progression [25–27]. Numerous studies demonstrate the crucial role of DNMT1 in various types of cancers, including acute myeloid leukemogenesis [28], breast cancer [29], prostate cancer [30], pancreatic cancer [30], lung cancer [31]. However, the role of DNMT1 in ESCC has not been investigated yet. In the current study, we report silencing DNMT1 inhibits proliferation, metastasis and invasion in ESCC cells, and suppresses tumor growth of ESCC in nude mice. Moreover, silencing DNMT1 induces G1 arrest and cell apoptosis in ESCC cells. Taken together, these data indicate silencing DNMT1 is a therapeutic target for ESCC therapy.

Previous studies demonstrates that DNMT1 mediated epigenetic silence of tumor related genes is the major mechanism contributes to carcinogenesis and progression of cancer. So, RNAi-mediated depletion of DNMT1, which results in CpG island demethylation and re-expression of tumor suppressor genes, may be a strategy for cancer therapy [32]. We have proved silencing DNMT is effective for inhibiting proliferation, colony formation, migration and invasion of ESCC cells. however, the molecular mechanism underlying inhibitory effects of silencing DNMT1 is not clear yet. In the current study, we focus on effects of silencing DNMT1 on expression and methylation status of DAPK, MGMT, RASSF1A, APC, ASC, P16 and CDH13, which has been reported frequently hypermethylated in ESCC. We found DAPK and RASSF1A were up-regulated in ESCC stable cells, in which DNMT1 was suppressed by shRNA. Similar results were also obtained in tumors isolated from nude mice, which were injected with ESCC stable cells. Moreover, methylation status of DAPK and RASSF1A were suppressed in ESCC stable cells and tumors isolated from nude mice. These data indicate that depletion of DNMT1 leads to suppression of methylation in CpG island and re-expression of DAPK and RASSF1A in ESCC.

In current study, expression of MGMT, APC, ASC and CDH13 in ESCC stable cells weren't increased by silencing DNMT1. One of the possible explanations is that methylation of tumor related genes is changed in different stage of cancers, including ESCC. e.g. methylation of p16 is a frequent and early event in ESCC [33], methylation of CDH13 is associated with high grade and advanced stage cancer [34, 35]. In vitro assays used in this study only reveal effects of silencing DNMT1 on methylation in one aspect, but cannot mimic the progression of ESCC. In vivo model which can present progression of ESCC hasn't been developed yet.

In conclusion, our data support the hypothesis that silencing DNMT1 inhibits proliferation, metastasis and invasion in ESCC by suppressing methylation of RASSF1A and DAPK. Although this work reveal the potential of DNMT1 as therapeutic target of ESCC, further investigation is needed to unveil the mystery about utilizing DNMT1 to develop tumor-oriented reagent for WSCC therapy.

## MATERIALS AND METHODS

### Cell culture

ESCC cell lines KYSE-150, KYSE-410 and KYSE-450 were obtained from Shanghai Cell Institute, (Shanghai, China). K150, K410 and K450 cells were grown in 1640 medium with 10% fetal bovine serum (FBS) (GIBCo/BRL, MD), supplemented with 100U/ml penicillin G and 100 μg/ml streptomycin (Sigma-Aldrich Corp., St. Louis, MO). Cells were maintained at 37°C in a humidified 5% CO2 incubator.

### Lentivirus production and transduction

Lentivirus expressing shRNA targeted DNMT1 were obtained from GenePharma Co., Ltd (China). To generate lentivirus, pWPXL-shRNA or pWPXL vector were transfected together with psPAX2 and pMDG2 into HEK293T cells using lipofectamine 2000 reagent. For transient infection, ESCC cells were infected with the recombinant lentivirus in the presence of 5 μg/mL Polybrene (Sigma). The impacts of of DNMT1-shRNA on ESCC cell lines were measured after transfected for 48h (Supplementary Figure S1).

### Creation of stable cells

ESCC cells were infected with recombinant lentivirus in the presence of 5 μg/mL Polybrene (Sigma). Infected cells were selected for 14 days in the presence of 2 μg/mL puromycin. (Sigma). Expression of DNMT1 in infected cells was verified by quantitative reverse transcription PCR (qRT-PCR) and western blot analysis. The expression of DNMT3a and DNMT3b in the stable-knockdown cells was detected by quantitative real time RT-PCR and western blot (Supplementary Figure S2).

### Quantitative real time RT-PCR (qRT-PCR)

Total RNA was extracted using TRIZOL reagent (Ambion) according to manufacturers' protocols. cDNA used to examined expression of DNMT1, RASSF1A and DAPK were synthesized by using PrimeScriptTM RT reagent kit (TaKaRa) according to manufacturers' protocols. Expression of DNMT1, RASSF1A and DAPK was examined using SYBR® Premix Ex TaqTM II (TaKaRa) and GAPDH was served as internal reference. All experiments were performed in duplicate and repeated twice. Results are represented as fold induction using the 2-ΔΔCt method. Primers used to examined expression of DNMT1, RASSF1A and DAPK were listed in Table 1.

**Table 1:** primers for quantitative real-time RT-PCR

ID	Sequence(5′- 3′)
CDH13 F1	AGTGTTCCATATCAATCAGCCAG
CDH13 R1	CCTTACAGTCACTGAAGGTCAAG
MGMT F1	ACCGTTTGCGACTTGGTACTT
MGMT R1	GGAGCTTTATTTCGTGCAGACC
APC F1	TCCTGTCCCTGTATCAGAGACT
APC R1	ACTGTGTTTGCTTGAGCTGCT
ASC F1	AGAATTGATGGCCGGAATTACAG
ASC R1	TGGAGCCTTGATAGAAGTCCTC
DNMT1 F1	CCTCTATGGAAGGCTCGAGT
DNMT1 R1	TCACCACACGGTGCTGCTCT
GAPDH F	ACACCCACTCCTCCACCTTT
GAPDH R	TTACTCCTTGGAGGCCATGT
DAPK F	TGGATATGACAAAGACACATC
DAPK R	CTTCATGTCCTTTGACCCAGA
RASSF1A F	ACAGCAACCTCTTCATGAGCT
RASSF1A R	CAAGGAGGGTGGCTTCTTGCT
p16 F	TGAAAGAACCAGAGAGGCTCT
p16 R	TGTAGGACCTTCGGTGACTGAT

### Western blot analysis

Western blot analysis was performed according to standard Western blot procedures as previously described. Briefly, proteins were separated by 10% SDS-PAGE and then transferred to nitrocellulose membrane (Bio-Rad). After blocking in 5% nonfat milk, the membranes were incubated with the following primary antibodies: mouse anti-DNMT1 monoclonal antibody (mAb; 1:300; Abcam), mouse anti-DAPK monoclonal antibody (mAb; 1:300; ABGENT), mouse anti-RASSF1A monoclonal antibody (mAb; 1:300; ABGENT), mouse anti-GAPDH mAb (1:1,000; ABGENT). The proteins were visualized with enhanced chemiluminescence reagents (Pierce).

### Proliferation assays

Cell Counting Kit-8 (CCK8) was used evaluate cell growth of ESCC cells according to the manufacturer's protocol. Briefly, 1×104 cells/well were plated in triplicate in 96-well plates. The CCK8 solution was added to each well at a 1:10 dilution. Cells were incubated for 4 h, and the absorbance at 450 nm was measured using a multi-well plate reader.

### Colony formation assays

Colony formation assays was performed as previously described. Briefly, 100 cells/well were plated in triplicate in 6-well plates and cultured for one week to form colonies. Colonies were stained with crystal violet staining solution for 20 min, and then the number of colonies was recorded.

### Transwell assays

The assay was done by using chambers with polycarbonate filters (pore size, 8μm) (Becton Dickinson Labware). ESCC cells were harvested and 5×104 cells in 200μL of 0.1% serum medium were placed in the upper chamber. The lower chamber was filled with 10% fetal bovine serum medium (600 μL). After 24h incubation and removal of the cells on the upper chamber of the filter with a cotton swab, the cells on the underside were fixed with 4% paraformaldehyde, stained with 0.1% crystal violet in 20% ethanol, and counted in five randomly selected fields under phase contrast microscope. The migrated cells were monitored by photographing at 400× magnification with LEICA Microscope. The assays were performed in triplicate.

### Tumori genesis assays

Six-week-old female athymic nude mice were subcutaneously injected in the right armpit region with1×107 cells in 0.1 mL of PBS. Two groups of mice(n = 6/group) were tested. Group 1 (NC) was injected with ESCC cells infected with NC; and group 2 (shRNA) was injected with ESCC cells infected with lentivirus containing shRNA targeted DNMT1. The tumor sizes was measured every 7 days with calipers. The tumor volume was calculated with the formula: (L × W2)/2, where L is the length and W is the width of the tumor. After the mice were killed at five weeks, the weights of the tumors were measured. All experimental procedures involving animals were in accordance with the Guide for the Care and Use of Laboratory Animals (NIH publication no. 80-23, revised 1996) and were performed according to the institutional ethical guidelines for animal experiments.

### Cell cycle analysis

For cell cycle analysis, 2×105 cells were plated in a 6-well culture plate and grown for 24 h. Cells were trypsinized, washed twice with cold PBS and fixed with cold 70% ethanol at −4°C overnight. The cells were then washed twice with PBS and incubated with 10 mg/ml RNase A, 400 mg/ml propidium iodide (Sigma-Aldrich) and 0.1% Triton-X in PBS at room temperature (RT) for 30 min. Cells were subsequently analyzed by flow cytometry.

### Apoptosis assays

Annexin V-FITC apoptosis detection kit (MUTISCIENCES, China) was used to detect apoptosis in ESCC cells. According to manufacturer's instruction manual, the cells were digested with trypsin, and centrifuged on 2000rpm for 5min. After collection, the cells were washed twice with PBS, and centrifuged on 2000rpm for 5min, 1-5 × 105 cells were collected and suspensed with 500μl Binding Buffer. 5μl Annexin V-FITC and 5μl Propidium Iodide was respectively added and mixed on room temperature, and away from light for 15min. Within 1 hour, the cells were detected by flow cytometry.

### Immunohistochemistry staining

The tumors was isolated, post-fixed in cold 4% paraformaldehyde overnight, and embedded in paraffin. Transverse paraffin sections (5 μm thickness) were mounted in Poly-L-Lysine-coated slides for histopathological examination. The tumor sections stained with hematoxylin and eosin for HE staining following the instruction. Consecutive slides were immunostained. The slide were incubated in 3% H2O2 for 15 min and 80% carbinol for 30 min and then in blocking solution for 1 h at room temperature. Subsequently, the sections were incubated at 4°C overnight with the following primary antibodies: DAPK (1:150) and RASSF1A (1:200). After triple washing in PBS, the sections were incubated with horseradish peroxidase-conjugated secondary antibodies for 2 h at 37°C. The reaction was stopped with 3, 3-diaminobenzidine (DAB). The results were imaged at a magnification of 400 using a Nikon ECLPSE 80i (Nikon, Japan). The optical densities and positive cell numbers of DAPK and RASSF1A were counted at 5 randomly selected fields per sample in spinal anterior horn and quantification by Imagepro-Plus. Histology and immunohistochemistry for each marker were performed simultaneously in all samples as well as negative controls without primary antibodies.

### Methylation-specific polymerase chain reaction (MSP)

MSP was performed as previously described [6]. Genomic DNA (1 μg) was harvested with a DNA rapid extraction kit for bisulfite modification with the CpG genome kit according to the manufacturer's instructions; 5 μL of bisulfite-modified DNA was used per 25 μL methylation-specific PCR (MSP) reaction. For PCR, methylated (M) and unmethylated (U) primer pairs were initially denatured at 94°C for 3 min followed by 35 cycles with a 1-min denaturation step, 30 s of annealing at 60°C(demethylation 58°C), and 3 min of extension at 72°C. Final extension after 35 cycles was at 72°C for 10 min, and the product was stored at 4°C. PCR products were analyzed by agarose gel electrophoresis and ethidium bromide staining, and the objective gene stripes were used as the positive expression. The methylation-specific primers and the unmethylation-specific primers used in MSP were listed in Table 2.

**Table 2:** primers for MSP analysis

ID	Sequence(5′- 3′)
DNMT1-U-F	TTAGTAAATTGTGGAGTTTGGATGA
DNMT1-U-R	TACAAAAAAATAAAACAAAACCAAC
DNMT1-M-F	TTTTAGTAAATCGTGGAGTTTGGAC
DAPK-M-R	TACAAAAAAATAAAACGAAACCGAC
DAPK-U-F	GGAGGATAGTTGGATTGAGTTAATGTT
DAPK-U-R	CAAATCCCTCCCAAACACCAA
DAPK-M-F	GGATAGTCGGATCGAGTTAACGTC
DAPK-M-R	CCCTCCCAAACGCCGA
RASSF1A-U-F	GGGGTTTGTTTTGTGGTTTTGTTT
RASSF1A-U-R	AACATAACCCAATTAAACCCATACTTCA
RASSF1A-M-F	GGGTTCGTTTTGTGGTTTCGTTC
RASSF1A-M-R	TAACCCGATTAAACCCGTACTTCG

### Bisulphite sequences assay (BSP)

Bisulphite sequences assay was performed to demonstrate the methylation status of RASSF1A and DAPK promoters in three ESCC stable cell lines (K150-shRNA, K410-shRNA and K450-shRNA). Genomic DNA from cell lines was isolated with Easypure Genomic DNA kit (TransGen Biotech, Beijing, China). Genomic DNA was subjected to bisulfite conversion and purification using the EZ DNA Methylation-GoldTM Kit (Zymo Research Corporation, CA, USA) according to the manufacturer's instructions. The BSP primer (Supplementary Table S1) was designed by Methprimer or Methyl Primer Express v1.0. Amplified PCR Products were purified and cloned into pMD19-T (TaKaRa, Dalian, China), 5 clones each cell were sequenced. Percentage of methylation was calculated comprehensively and comparatively by CpG viewer, QUMA and Biq-analyzer. The primer sequence for BSP were listed in table 3.

**Table 3:** The primer sequence for BSP in this reasearch

primer	5′- 3′
DAPK-BSP-F	GAGGTTTTTAGTGGATATGGGATTT
DAPK-BSP-R	TCCACCTCCAAAATTCAAATAATT
RASSF1A-BSP-F	TTATTTAGTGGGTAGGTTAAGTGTGTT
RASSF1A-BSP-R	CCTAAATACAAAAACTATAAAACCC

### Statistical analysis

Data were shown as mean ± SD unless otherwise noted; the Student's t test was used to analyze statistical difference between group NC and group shRNA. P value of <0.05 was considered statistically significant.

## SUPPLEMENTARY FIGURES AND TABLE


