# Supplementary material for: Silencing DNA methyltransferase 1 (DNMT1) inhibits proliferation, metastasis and invasion in ESCC by suppressing methylation of RASSF1A and DAPK
Source: Oncotarget. 2016 Jun 7;7(28):44129–41. doi: 10.18632/oncotarget.9866 (PMC5190084; doi:10.18632/oncotarget.9866)
Supplement: Supplementary file 1 [file oncotarget-07-44129-s001.pdf]

Silencing DNA methyltransferase 1 (DNMT1) inhibits proliferation, metastasis and invasion in ESCC by suppressing methylation of RASSF1A and DAPK

SUPPLEMENTARY FIGURES AND TABLE

Supplementary Figure S1

To demonstrate the impacts of transient transfection of DNMT1-shRNA on ESCC cell lines, the mRNA and protein expression of DNMT1 were measured after transfected for 48h. The expression of DNMT1 at mRNA and protein lever were decreased significantly in K410, K450 and K150 cell lines (Supplementary Figure 1). This finding was consistent with results obtained from three ESCC stable cell lines--K150-shRNA, K410-shRNA and K450-shRNA(Figure 1 B&C).

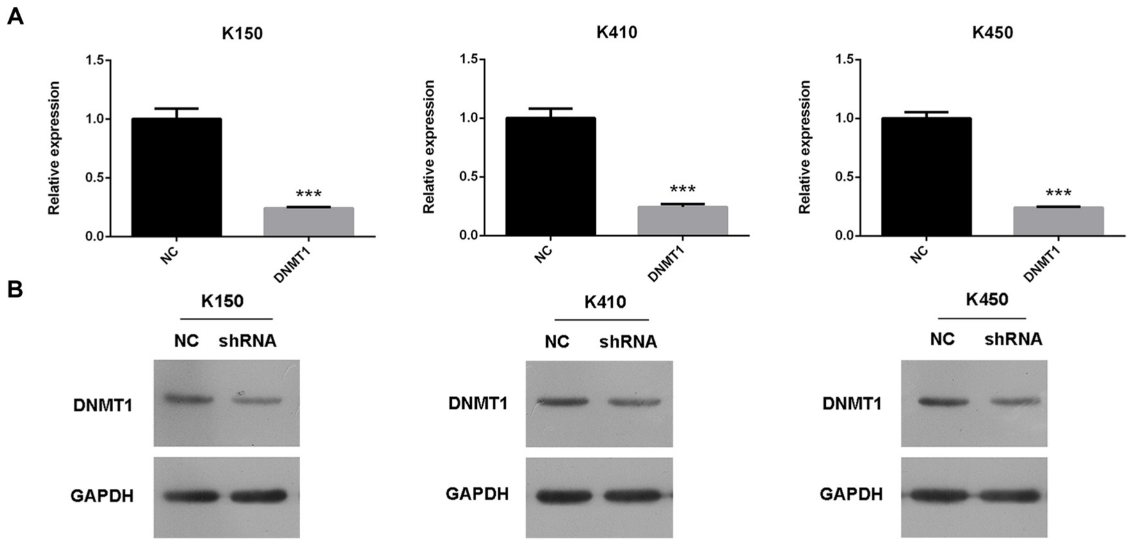

Supplementary Figure S1: Silencing DNMT1 in ESCC cells for 48h. A. The mRNA expression of DNMT1 in ESCC cells. mRNA expression of DNMT1 was normalized to GAPDH. Data represented as means ± SD from three independent experiments. ***, $p < 0.001$. **B.** The protein expression of DNMT1 in ESCC cells. GAPDH was served as loading control.

Supplementary Figure S2

The influence of DNMT1-shRNA on DNMT3a and DNMT3b was detected by western blot and quantitative real time RT-PCR (Supplementary Fig 2A&B). These results showed the expression of DNMT3a and DNMT3b in mRNA and protein levels were decreased (p < 0.05) or unchanged in three ESCC stable cell, which was not obviously compared to DNMT1 (p < 0.001). So, the DNMT1 was first selected for further study.

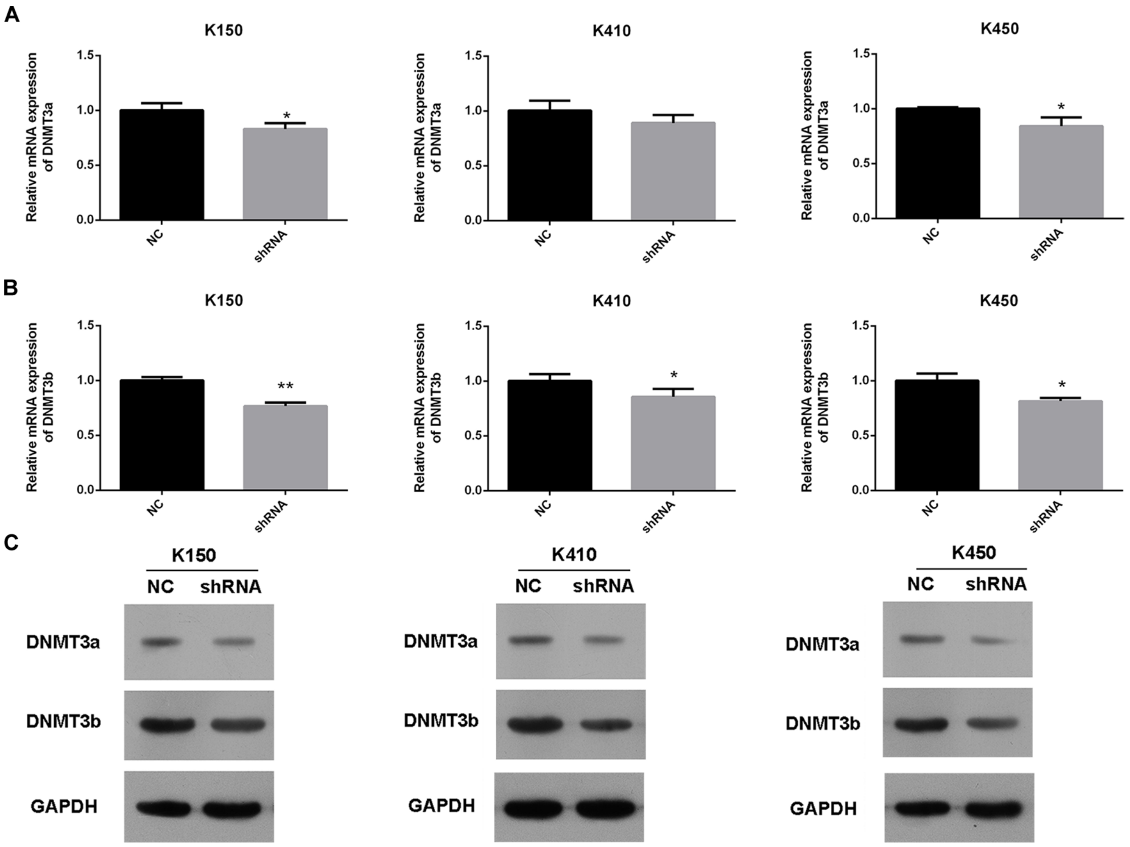

Supplementary Figure S2: The mRNA and protein expression of DNMT3a and DNMT3b in three ESCC stable cells. A&B. The mRNA expression of DNMT3a and DNMT3b in three ESCC stable cell, respectively. mRNA expression of DNMT1 was normalized to GAPDH. Data represented as means ± SD from three independent experiments. ***, p < 0.001. **C.** The protein expression of DNMT3a and DNMT3b in three ESCC stable cell. GAPDH was served as loading control.

Supplementary Figure S3

RASSF1A and DAPK were overexpressed in DNMT1-depleted cells and examined the cell proliferation and migration, respectively. Quantitative real time RT-PCR and western blot results showed that the mRNA and protein expression of RASSF1A and DAPK were significantly increased in DNMT1 knockdown cells, compared to the control group (Supplementary Figure 3).

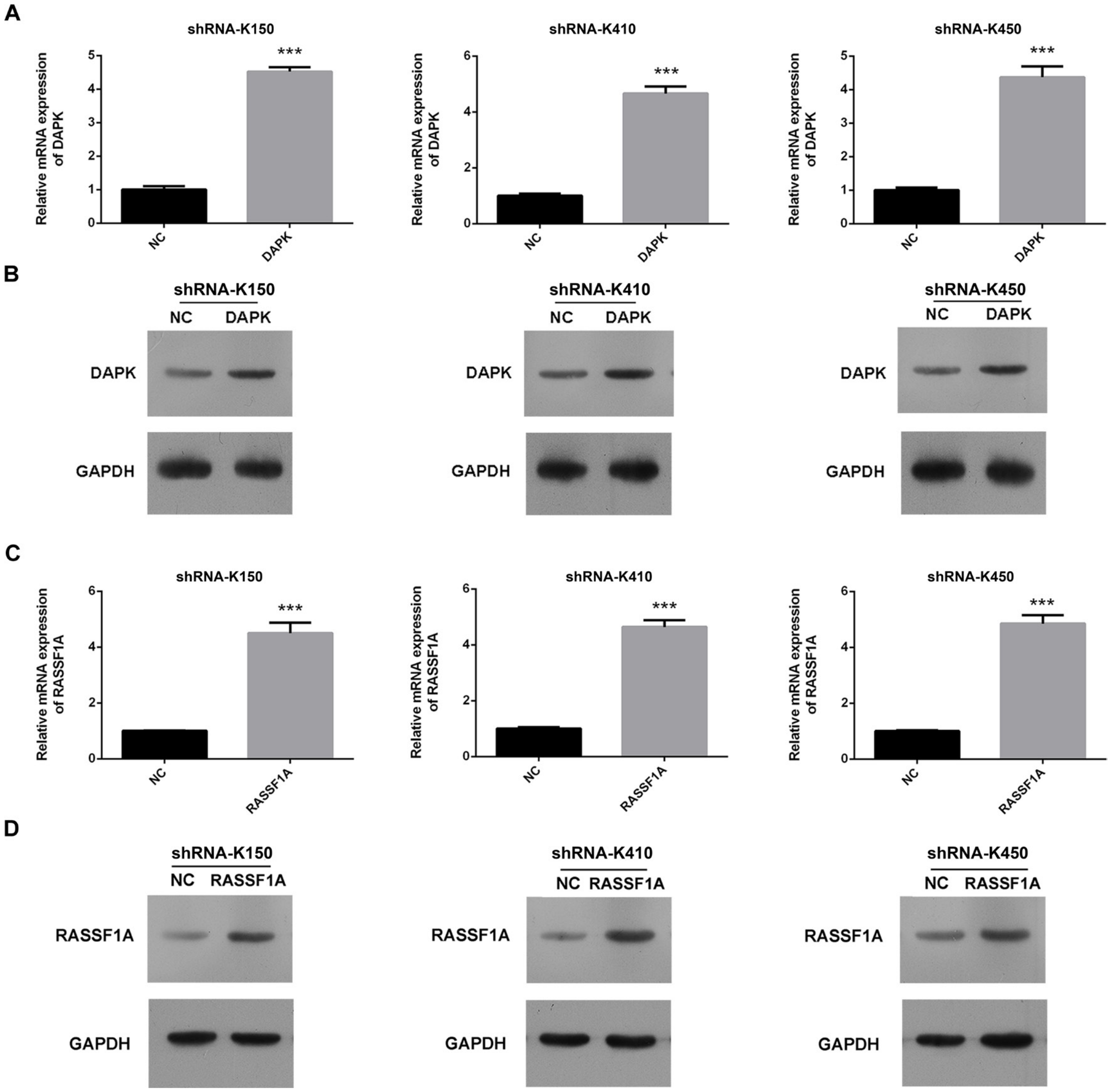

Supplementary Figure S3: Overexpress DAPK and RASSF1A in three ESCC stable cells. **A.** The mRNA expression of DAPK in three ESCC stable cells. **B.** The mRNA expression of RASSF1A in three ESCC stable cells. mRNA expression of DNMT1 was normalized to GAPDH. Data represented as means ± SD from three independent experiments. ***, $p < 0.001$. **C.** The protein expression of DAPK in three ESCC stable cells. GAPDH was served as loading control. **D.** The protein expression of RASSF1A in three ESCC stable cells. GAPDH was served as loading control.

Supplementary Figure S4&S5

Cell proliferation assays and transwell assays showed that overexpressing RASSF1A or DAPK markedly inhibits proliferation, metastasis and invasion in three ESCC stable cell lines (K150-shRNA, K410-shRNA and K450-shRNA). This finding were is more obvious than that for silencing DNMT1 (Figure 2 A&D).

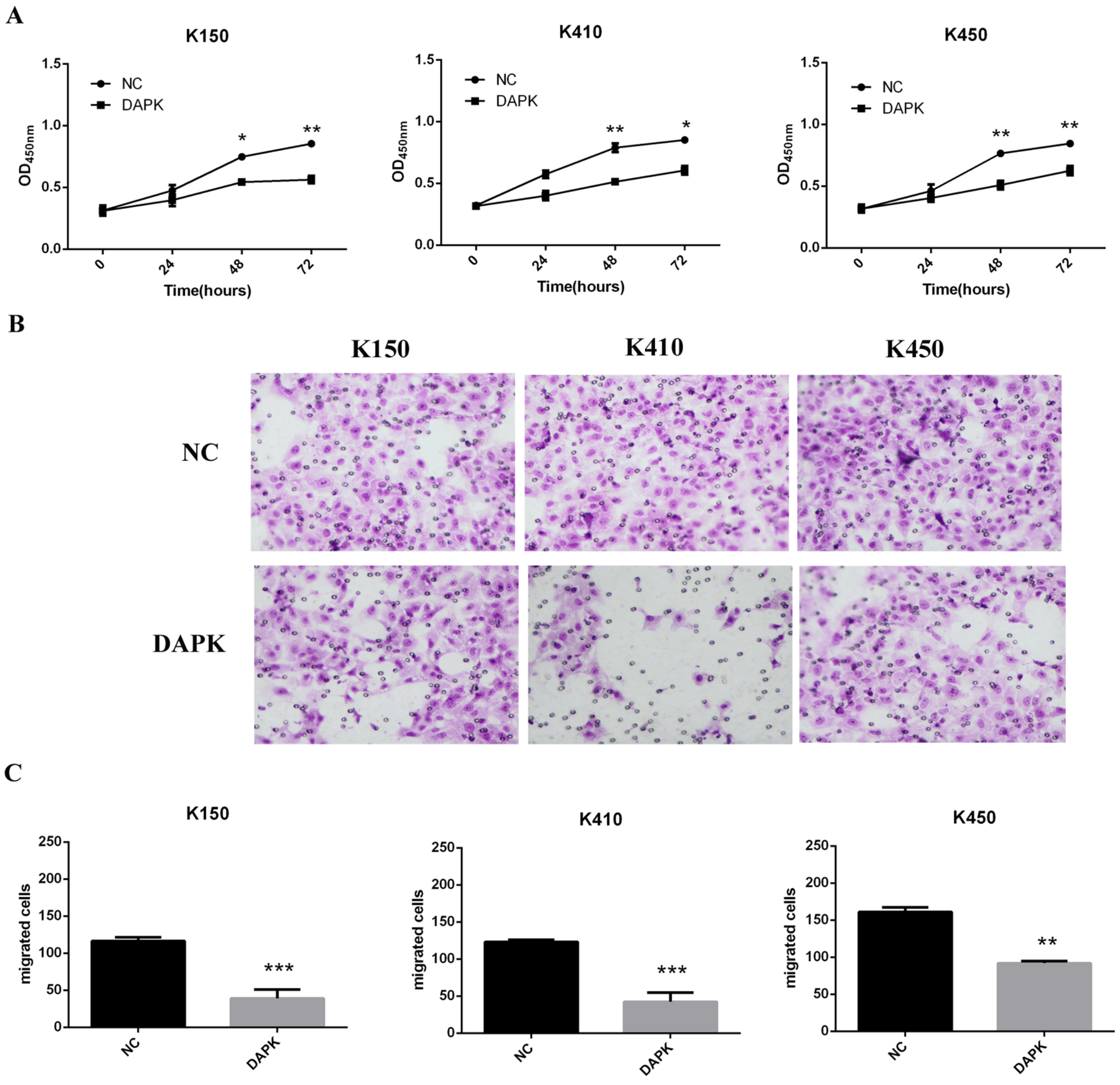

Supplementary Figure S4: Overexpress DAPK inhibited proliferation and invasion in three ESCC stable cells. A. MTT assays of three ESCC stable cells. Data represented as means ± SD from three independent experiments. *, $p < 0.05$. **B&C.** invasion assays of three ESCC stable cells. original magnification, 20 ×. Data represented as means ± SD from three independent experiments. ***, $p < 0.001$.

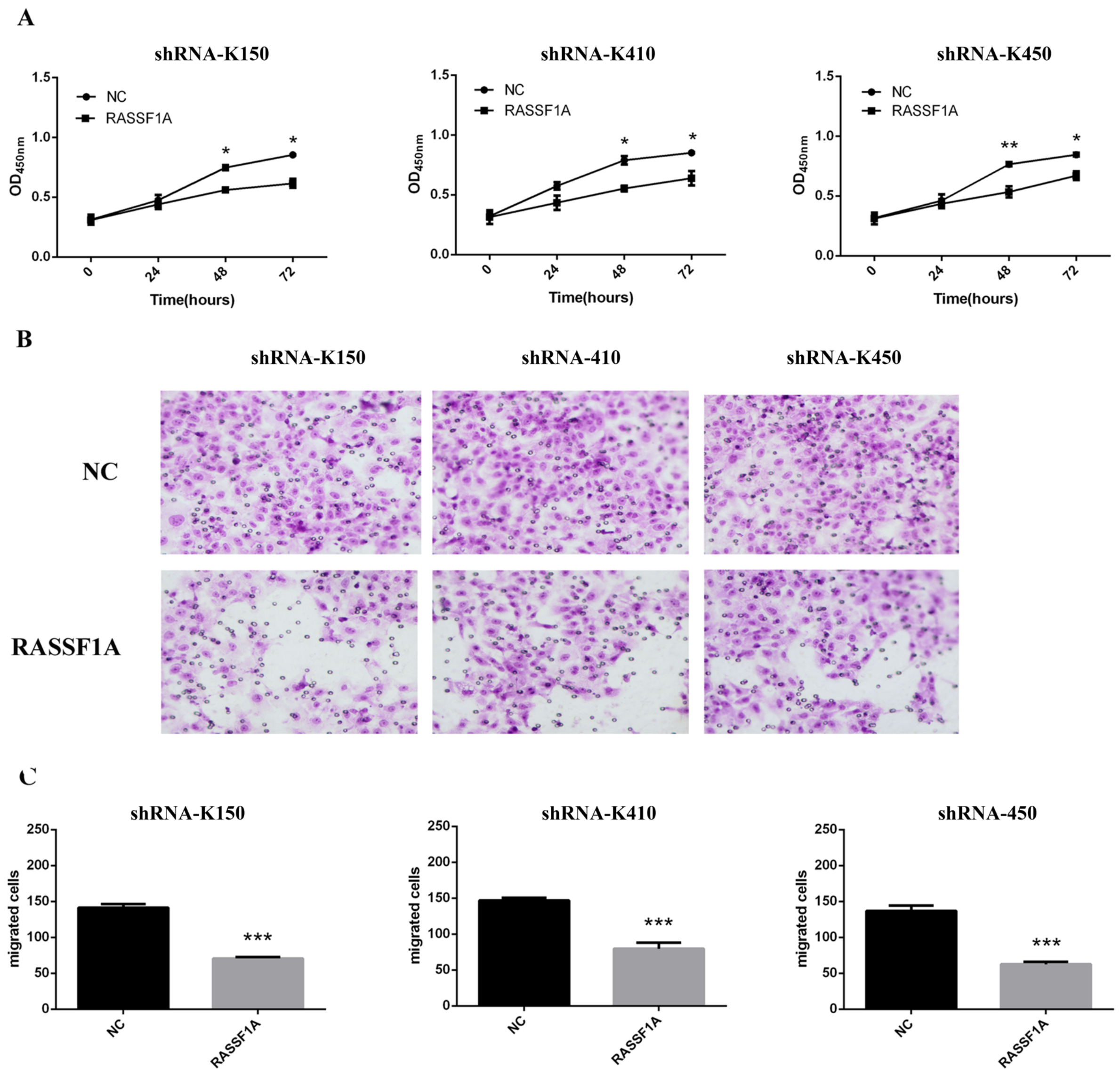

Supplementary Figure S5: Overexpress RASSF1A inhibited proliferation and invasion in three ESCC stable cells. A. MTT assays of three ESCC stable cells. Data represented as means ± SD from three independent experiments. *, $p < 0.05$. **B&C.** invasion assays of three ESCC stable cells. original magnification, 20 ×. Data represented as means ± SD from three independent experiments. ***, $p < 0.001$.

Supplementary Table S1: The primer sequence for gene overexpression in this reasearch

primer	5’- 3’
DAPK- F	CGGAATTCATGATTACTACGACACCGGCGA
DAPK- R	AAATATGCGGCCGCTCACCGGGATACAACAGAGCTA
RASSF1A -F	CGGGATCCATGTCGGGGGAGCCTGAGCTCATTG
RASSF1A -R	AAATATGCGGCCGCTCACCCAAGGGGGCAGGCGTG
